# A modified predator–prey model for the interaction of police and gangs

**DOI:** 10.1098/rsos.160083

**Published:** 2016-09-14

**Authors:** J. Sooknanan, B. Bhatt, D. M. G. Comissiong

**Affiliations:** 1 Centre for Education Programmes, The University of Trinidad and Tobago, Trinidad, West Indies; 2 Department of Mathematics and Statistics, The University of the West Indies, St. Augustine, Trinidad, West Indies

**Keywords:** predator–prey, crime model, Beddington–De Angelis

## Abstract

A modified predator–prey model with transmissible disease in both the predator and prey species is proposed and analysed, with infected prey being more vulnerable to predation and infected predators hunting at a reduced rate. Here, the predators are the police and the prey the gang members. In this system, we examine whether police control of gangs is possible. The system is analysed with the help of stability analyses and numerical simulations. The system has five steady states—four of which involve no core gang members and one in which all the populations coexist. Thresholds are identified which determine when the predator and prey populations survive and when the disease remains endemic. For parameter values where the spread of disease among the police officers is greater than the death of the police officers, the diseased predator population survives, when it would otherwise become extinct.

## 1. Introduction

The use of mathematics as an alternative weapon in the fight against crime is on the rise. Though mathematics has been traditionally associated with the statistical analysis of crime data and statistical models of crime and criminal behaviour [1,2], the development of non-statistical mathematical models constructed from agent-based models, predator–prey models and infectious disease models has been increasing due to advances in numerical analysis and computers.

Agent-based models are popular in investigating the spatio-temporal dynamics of crime. According to Malleson & Evans [3], they are well suited to modelling crimes ‘which are heavily influenced by environmental factors and by the behavior of individual people’. They have been used to study the mapping of crime hotspots and the displacement of crime [4], the efficacy of different policing strategies on the suppression of crime [5], the dynamics of crime hotspots [6] and the relationship between crime and punishment [7]. An interesting development in agent-based modelling is the incorporation of information from geographical information systems (GISs) so as to make the model more realistic. Such models have been used to study street gang rivalries [8], to examine routine activity theory as it applies to street robbery [9] and to test different crime prevention strategies [10]. Generally, in applying these models to crime and criminal behaviour, the agents represent people—criminals, potential victims, police, etc. These agents inhabit an artificial environment that is designed to reflect features such as buildings, a street network, a social network, or barriers to movement. Their movement and interaction may be regulated by either equations or ‘behavioural rules and responses to environmental information’ [11] that scientists, social scientists and police would expect to see in real life. A comprehensive review of these models may be found in D’Orsogna & Perc [12].

The social nature of some types of crime and criminal behaviour is the basis of models adapted from population biology such as infectious disease models and predator–prey models, both of which deal with interacting groups. ‘From a mathematical point of view these groups interact in a way remarkably similar to those that are common in population biology’ [13]. Infectious disease models have been applied to violent crime and burglary in the UK, where criminal behaviour was treated as a socially infectious disease [14,15]. A similar analogy was used for gangs [16,17], where gang membership is treated as an infection that multiplies due to interaction or peer contagion by ‘infected’ youth with intervention and prevention measures included in the model.

Another model common in population biology, the predator–prey model, has been used to describe the interactions between police officers (predators) and criminals (prey) and the corresponding effects of changes in policy and law enforcement [18]. Other modelling efforts include criminals treated as predators preying upon two groups of people who band together in group defence [19] and a predator–prey system consisting of owners *X* who are the prey, criminals *Y* who are the predators of *X*, and the security guards *Z* are predators of both *X* and *Y* [20].

The model developed in this paper examines the police–gang relationship where police officers act as predators of gang members and may become corrupted by them resulting in criminal behaviour on their part. Internationally, there is widespread evidence of the pervasiveness of police corruption [21]. These corrupt practices range from bribery and extortion to police involvement in criminal activities [22]. We model the dynamics of the police–gang relationship using a combination of an infectious disease and a predator–prey model from population biology also known as an eco-epidemiological model.

Our goal is to give a general description of how gang membership responds to various crime fighting strategies and policy changes and to identify ‘tipping points’ which may result in the disappearance of gangs and corrupt police officers from the population. The behaviour of the model is investigated through stability and bifurcation analysis. The paper is organized as follows: §2 contains a description of the model and its inherent assumptions. The different equilibria and their stability are analysed in §3, with the bifurcation analysis and discussion in §4.

## 2. Material and methods

### 2.1. The mathematical model

The model uses the analogy of police officers as predators of gang members. Also, gang membership is treated as an infection that is spread to police officers by both gang members and other police officers. Hence, two populations are considered: the gang population, *N*_g_, and the police population, *N*_p_. The gang population is divided into two compartments: *S*_g_ and *I*_g_—based on their commitment to the gang [23]. *S*_g_ contains susceptible gang members, who are not yet fully committed to the gang lifestyle. The population *I*_g_ contains the committed core gang members.

The predators of the gangs, the police officers, are divided into those who function within the police service yet have affiliations to gangs, corrupt police officers (*I*_p_), and susceptible police officers (*S*_p_) who are fully committed to the police service. The spread of the infection is described by a standard incidence contact term—this form best describes large populations with a low number of infected individuals [24].

Youth who are not fully committed to the gang (*S*_g_) may by contact rate (*β*_1_) with the committed gang members (*I*_g_) join the gang at a rate of *β*_1_*S*_g_*I*_g_/*N*_g_. However, the gang members are being sought or hunted by the police officers at a rate described by the functional responses *f*_1_(*N*_g_,*N*_p_)*N*_p_ and *f*_2_(*N*_g_,*N*_p_)*N*_p_ depending on their gang membership status. When captured by the police officers, they go into the criminal justice system *Z*. There is a growth rate *b* for the susceptible population and a removal rate *μ*_1_ for both gang populations. In-fighting among gang members is expressed as intraspecific competition with the forms (*S*_g_*N*_g_/*K*) and (*I*_g_*N*_g_/*K*), where *K* is related to the maximum population abundance K* an environment can sustain: *K*=K*/(*b*−*μ*_1_).

Susceptible police officers may become corrupt in two ways—by interaction with the committed gang members at a rate (*β*_2_*S*_p_*I*_g_/*N*_p_) and by interaction with corrupt police officers at a rate (*β*_3_*S*_p_*I*_p_/*N*_p_). However, the corrupted police officers will be ‘hunted’ by susceptible police officers described by a functional response *g*__1__(*N*_g_,*N*_p_)*S*_p_. When captured by the police officers, they go into the criminal justice system *Z*. There is a growth rate *γ* and a removal rate *μ*_2_ for the police officer populations. All these parameters are non-negative because negative parameters have no real-life significance.

#### 2.1.1. Assumptions

To formulate and analyse the model, the following assumptions were made:

(i) The definition of corruption in this model specifically refers to corruption of police officers by gang members.
(ii) Individuals in the gang and police officer populations are either susceptible (when they can become corrupted) or infectious (when they are corrupted). Here, we assume that the infection is lifelong. This corresponds to an SI (susceptible infectious) infectious disease model.
(iii) Disease does not spread from police officers to the gang members. All police officers are susceptible to criminality/being corrupted by gang members, but not vice versa, i.e. the corrupt police officers do not recruit gang members or other criminals.
(iv) We assume that once criminals are caught by the police officers, they enter the criminal justice system *Z* and do not re-enter the population so that *Z* acts as a sink in the system.
(v) The susceptible police officers have a greater ‘hunting ability’ than corrupt police officers. The committed core gang members are more vulnerable to being arrested by the police officers.
(vi) The detection and arresting of gang members translates to the hunting and handling time to find, capture and consume prey.
(vii) As countries generally have a target number of police officers, we assume that the total number of police officers *N*_p_=*S*_p_+*I*_p_=*P* in the system is constant.
(viii) The rate at which persons join the police force, *γ*, is equal to the rate at which they leave it, *μ*_2_.
(x) Discrete and stochastic effects are not considered.

The model describing the relations between the state variables is:

$$\begin{array}{rl} \frac{dS_{g}}{dt} & =bN_{g}-\frac{S_{g}N_{g}}{K}-\frac{\beta_{1}S_{g}I_{g}}{N_{g}}-f_{1}(N_{g},N_{p})N_{p}-\mu_{1}S_{g}\\ \frac{dI_{g}}{dt} & =\frac{\beta_{1}S_{g}I_{g}}{N_{g}}-\frac{I_{g}N_{g}}{K}-f_{2}(N_{g},N_{p})N_{p}-\mu_{1}I_{g}\\ \frac{dS_{p}}{dt} & =\gamma N_{p}-\frac{\beta_{2}S_{p}I_{g}}{N_{p}}-\frac{\beta_{3}S_{p}I_{p}}{N_{p}}-\mu_{2}S_{p}\\ \frac{dI_{p}}{dt} & =\frac{\beta_{2}S_{p}I_{g}}{N_{p}}+\frac{\beta_{3}S_{p}I_{p}}{N_{p}}-g_{{\;}_{1}}(N_{g},N_{p})S_{p}-\mu_{2}I_{p}\\ \frac{dZ}{dt} & =f_{1}(N_{g},N_{p})N_{p}+f_{2}(N_{g},N_{p})N_{p}+g_{{\;}_{1}}(N_{g},N_{p})S_{p}-\mu_{3}Z\\ N_{g} & =S_{g}+I_{g}\\ N_{p} & =S_{p}+I_{p}=P \end{array}$$

with initial conditions *S*_g_(0)≥0,*I*_g_(0)≥0,*S*_p_(0)≥0,*I*_p_(0)≥0,*Z*(0)≥0.

The model can be shown to be mathematically well posed in the positively invariant region

$$G=\{(S_{g},I_{g},S_{p},I_{p},Z)|0\leq S_{g},I_{g}\leq K,0\leq S_{p},I_{p}\leq P,Z\geq 0\}$$

and solutions in *G* exist for all positive time.

### 2.2. Functional responses used in this model

The functional response of a predator refers to the relationship between the rate of prey consumption per predator and the prey density [25]. In this model, there are two functional response terms: *f*_1_(*N*_g_,*N*_p_) and *f*_2_(*N*_g_,*N*_p_) refer to the functional response terms of police officers who are hunting gang members.

#### 2.2.1. *f*_1_(*N*_g_,*N*_p_) and *f*_2_(*N*_g_,*N*_p_): functional response of police officers who are hunting criminals

To determine the functional response of the police officers, it is important to characterize the way in which the police officers interact with one another. It is assumed that the interaction among the police officers is similar to that of predators in nature. When the predation rate is dependent on the densities of both predator and prey, the model is termed predator-dependent. The following behaviours may lead to predator dependence: ‘group hunting; anti-predator defense by the prey; density dependent and time-consuming social interactions among the predators; aggressive interactions between searching predators that encounter each other and a limited number of high-quality sites where predators capture prey rapidly’ [26].

According to Skalski & Gilliam [27], three predator-dependent functional responses, the Beddington–De Angelis, the Crowley–Martin and the Hassell–Varley, can provide a description of predator feeding over a range of predator and prey abundance. They suggested using either the Beddington–De Angelis or Hassell–Varley model when the predator feeding rate was independent of predator density at high prey density. The Beddington–De Angelis functional response was chosen for its analytical tractability.

The Beddington–De Angelis functional response considers a sole predator. We, however, consider police officers working together in groups/teams of size *U*. The derivation below is based on the model by Beddington [28]. The time spent in hunting the gang members may be divided into: search, encounter, the choice to attack or not, capture and handling. Hence, the total time of a police operation

$$T=T_{s}+T_{h}-T_{c},$$

where *T*_s_ is search time; *T*_h_, handling time; *T*_*c*_, time saved due to pre-planning, cooperation and communication among police officers; *a*_g_*T*_s_*I*_g_, number of *I*_g_ captured per police team; *a*_s_*T*_s_*S*_g_, number of *S*_g_ captured per police team. The constants *a*_g_,*a*_s_ represent the attack rate of the police team or the rate of successful search also known as the encounter rate between predators and prey (i.e. the probability of a foraging predator encountering a prey item in one unit of time, given that the predator is searching throughout that time).

Let *t*_h_ represent the handling time per criminal. Handling time includes time for digestive pause before the next attack. It is divided into ingestion and digestion i.e. time for chasing, killing, eating and digesting each prey item. This corresponds to the time for capturing, subduing and processing each criminal, before they enter the criminal justice system. We assume that the handling time for *I*_g_ is greater than the handling time for *S*_g_ by *λ*. Therefore, the handling time *T*_h_ for *I*_g_=*λa*_g_*T*_s_*I*_g_*t*_h_ and the handling time *T*_h_ for *S*_g_=*a*_s_*T*_s_*S*_g_*t*_h_. Let *N*_e_ be number of cooperative encounters among police officers; *N*_p_, number of police, *S*_p_+*I*_p_; *b*^′^, rate of communication of information per police officers per unit search time; *t*_c_, time saved per encounter per police officer.

It follows that

$$N_{e}=b^{\prime }T_{s}N_{p}.$$

and

$$T_{c}=b^{\prime }T_{s}N_{p}t_{c}.$$

In the Beddington–De Angelis derivation, *N*_p_−1 was used as each predator would encounter *N*_p_−1 other police officers. As the operation is pre-planned, each police officer himself would save time because his actions and duties are known before, thus we use *N*_p_.

Recall that *T*=*T*_s_+*T*_h_−*T*_c_. Substituting in this equation, we get

$$T=T_{s}(1+t_{h}(a_{s}S_{g}+\lambda a_{g}I_{g})-b^{\prime }N_{p}t_{c}).$$

As gang members tend to stay together, let us assume that *a*_s_=*a*_g_=*a*. Therefore,

$$T_{s}=\frac{T}{1+at_{h}(S_{g}+\lambda I_{g})-b^{\prime }N_{p}t_{c}}.$$

Let us assume that police officers are divided into teams of size *U*, so *aT*_s_*I*_g_, refers to the number of gang members captured due to a team effort. Then, *G* is number of *I*_g_ captured per police officer, *aI*_g_*T*/*U*(1+*at*_h_(*S*_g_+*λI*_g_)−*b*^′^*N*_p_*t*_c_) and *S* is number of *S*_g_ captured per police officer, *aS*_g_*T*/*U*(1+*at*_h_(*S*_g_+*λI*_g_)−*b*^′^*N*_p_*t*_c_). Hence,

$$\begin{array}{rl} f_{1}(N_{g},N_{p}) & =\frac{aS_{g}}{U(1+h(S_{g}+\lambda I_{g})-cN_{p})}\\ \mathrm{and}\;f_{2}(N_{g},N_{p}) & =\frac{aI_{g}}{U(1+h(S_{g}+\lambda I_{g})-cN_{p})}, \end{array}$$

where *h*=*at*_h_ and *c*=*b*^′^*t*_c_. Descriptions of the parameters are shown in tables 1 and 2.

**Table 1.** Description of parameters used in the model.

parameter	description
*b*	entry rate into the gang
*γ*	number of trainees who graduate from the police academy per year
*μ*_1_	exit rate from gang
*μ*_2_	rate at which police officers leave service
*μ*_3_	rate at which people processed and go to jail/exonerated
*β*_1_	recruitment rate of youth into gang as committed members
*β*_2_	recruitment rate of susceptible police officers into gang by gang members
*β*_3_	recruitment rate of susceptible police officers by corrupt police officers
*f*_*i*_(*N*_g_,*N*_p_)	functional response of police officers who are hunting criminals (*i*=1,2)

**Table 2.** Definition of parameters in the Beddingtion–De Angelis functional responses.

parameter	description
*a*	rate at which police officers find criminals
*U*	size of police teams
*r*	corrupted police officers hunt at a reduced rate *r*
*t*_c_	cooperation time per police officer
*λ*	the handling time for *I*_g_ is greater than the handling time for *S*_g_ by *λ*
*t*_h_	handling time of each gang member
*b*′	rate of communication among police officers

### 2.3. The system

The model may be simplified by making the assumption that the number of police officers is constant so *N*_p_=*P*= constant. This reduces the system to four unknowns — *S*_g_,*I*_g_,*I*_p_,*Z*. We further assumed that corrupted police officers hunt at a reduced rate *r*. The governing equations become

2.1 $$\begin{array}{rl} & b(S_{g}+I_{g})-\frac{S_{g}(S_{g}+I_{g})}{K}-\frac{\beta_{1}S_{g}I_{g}}{N_{g}}-\frac{aS_{g}(P-I_{p}+rI_{p})}{U(1+h(S_{g}+\lambda I_{g})-cP)}-\mu_{1}S_{g}=S_{g}^{{\;}^{\prime }}, \end{array}$$

2.2 $$\begin{array}{rl} & \frac{\beta_{1}S_{g}I_{g}}{N_{g}}-\frac{I_{g}(S_{g}+I_{g})}{K}-\frac{aI_{g}(P-I_{p}+rI_{p})}{U(1+h(S_{g}+\lambda I_{g})-cP)}-\mu_{1}I_{g}=I_{g}^{{\;}^{\prime }}, \end{array}$$

2.3 $$\begin{array}{rl} & \frac{\beta_{2}(P-I_{p})I_{g}}{P}+\frac{\beta_{3}(P-I_{p})I_{p}}{P}-\mu_{2}I_{p}=I_{p}^{{\;}^{\prime }}, \end{array}$$

2.4 $$\begin{array}{rl} & \frac{aS_{g}(P-I_{p}+rI_{p})}{U(1+h(S_{g}+\lambda I_{g})-cP)}+\frac{aI_{g}(P-I_{p}+rI_{p})}{U(1+h(S_{g}+\lambda I_{g})-cP)}-\mu_{3}Z=Z^{{\;}^{\prime }}, \end{array}$$

2.5 $$\begin{array}{rl} \mathrm{and}\; & (b-\mu_{1})(S_{g}+I_{g})-\frac{{\left(S_{g}+I_{g}\right)}^{2}}{K}=N_{g}^{{\;}^{\prime }},\\ & N_{g}=S_{g}+I_{g},\\ & P=S_{p}+I_{p},\\ & N=N_{g}+P+Z. \end{array}$$

The equilibrium values can be determined by taking the derivatives of equations (2.1)–(2.5) to be zero.

## 3. Equilibrium values

### 3.1. *I*_g_=0: no core gang members

This represents possible states of the dynamical system in which all the core gang members can be considered eradicated. Substituting *I*_g_=0 results in four distinct equilibria. These are:

*E*_0_- all the gang members and the corrupt police officers become extinct,
*E*_1_- all the gang members become extinct and the disease remains endemic in the police force,
*E*_2_- both the committed core gang members and the corrupt police officers become extinct,
*E*_3_- core gang members become extinct, and the disease remains endemic in the police force.
  $$\begin{array}{rl} & E_{0}\text{: }S_{g}=0,I_{g}=0,I_{p}=0,Z=0\text{ trivial equilibrium}\\ & E_{1}\text{: }\left\{\begin{array}{c} S_{g}=0,\;I_{g}=0,\;Z=0,\\ I_{p}=\frac{P(\beta_{3}-\mu_{2})}{\beta_{3}},\text{ where }\beta_{3}>\mu_{2} \end{array}\right\}\begin{array}{c} \text{criminal-free}\\ \text{equilibrium} \end{array} \end{array}$$

The no-criminals equilibria — *E*_0_ and *E*_1_.

$$\begin{array}{rl} & E_{2}\text{: }\left\{\begin{array}{c} (b-\mu_{1})-\frac{S_{g}}{K}-\frac{aP}{U(1+hS_{g}-cP)}=0,\\ I_{g}=0,\;I_{p}=0,\\ \mu_{3}Z=(b-\mu_{1})S_{g}-\frac{S_{g}^{2}}{K} \end{array}\right\}\begin{array}{c} \text{core gang-free and}\\ \text{corrupted police-free}\\ \text{equilibrium} \end{array}\\ & E_{3}\text{: }\left\{\begin{array}{c} (b-\mu_{1})-\frac{S_{g}}{K}-\frac{a(P-I_{p}+rI_{p})}{U(1+hS_{g}-cP)}=0,\\ I_{g}=0,\;I_{p}=\frac{P(\beta_{3}-\mu_{2})}{\beta_{3}},\text{ where }\beta_{3}>\mu_{2}.\\ \mu_{3}Z=(b-\mu_{1})S_{g}-\frac{S_{g}^{2}}{K} \end{array}\right\}\begin{array}{c} \text{core gang-free}\\ \text{equilibrium} \end{array} \end{array}$$

The no-committed gang members equilibrium — *E*_2_ and *E*_3_.

Details on calculating the term *S*_g_ in the equilibria *E*_2_ and *E*_3_ are provided in appendix A.

### 3.2. *E*_4_: The coexistence equilibrium

The coexistence equilibrium (${\tilde{S\;}\;\;}_{g},{\tilde{I\;}\;\;}_{g},{\tilde{I\;}\;\;}_{p}$) is found from

3.1 $$\begin{array}{rl} {\tilde{I\;}\;\;}_{g} & =\left(\frac{\mu_{2}P-\beta_{3}(P-{\tilde{I\;}\;\;}_{p})}{\beta_{2}(P-{\tilde{I\;}\;\;}_{g})}\right){\tilde{I\;}\;\;}_{p}=f({\tilde{I\;}\;\;}_{p}),\\ \mathrm{and}\;{\tilde{S\;}\;\;}_{g} & =\left(\frac{b}{\beta_{1}-b}\right)\left(\frac{\mu_{2}P-\beta_{3}(P-{\tilde{I\;}\;\;}_{p})}{\beta_{2}(P-{\tilde{I\;}\;\;}_{p})}\right)=f({\tilde{I\;}\;\;}_{p}). \end{array}\}$$

Our analysis leads to a polynomial of the form

3.2 $$b_{1}I_{p}^{4}+b_{2}I_{p}^{3}+b_{3}I_{p}^{2}+b_{4}I_{p}+b_{5}=0,$$

the coefficients of which are provided in appendix B.

### 3.3. Stability of equilibria

To check the stability of the equilibrium points, we linearize the system by taking a small perturbation about the equilibrium points by substituting

3.3 $$S_{g}=S_{g}^{*}+u,\;I_{g}=I_{g}^{*}+v,\;I_{p}=I_{p}^{*}+w,\;Z=Z^{*}+y,$$

where *u*,*v*,*w*,*y* are small perturbations, and $S_{g}^{*},\;I_{g}^{*},\;I_{p}^{*},\;Z^{*}$ are equilibrium values. We expand all terms about the equilibria using Taylor’s theorem and neglect higher-order terms in *u*,*v*,*w*,*y*. The related eigenvalues are given by

3.4 $$\lambda^{3}+a_{1}\lambda^{2}+a_{2}\lambda +a_{3}=0,$$

the coefficients of which are provided in appendix C.

#### 3.3.1. Case *E*_0_:*S*_g_=0, *I*_g_=0, *I*_p_=0, *Z*=0: trivial equilibrium

On substituting *E*_0_ in *Q*, the eigenvalues are:

(i) −*μ*_2_<0,
(ii) $-\frac{a^{\prime }P}{\left(1-cP\right)}-\mu_{1}<0$ where $a^{\prime }=\frac{a}{U}$ and
(iii) $b-\frac{a^{\prime }P}{\left(1-cP\right)}-\mu_{1}.$

Hence, the condition for stability for the trivial equilibrium is

3.5 $$\frac{a^{\prime }P}{\left(1-cP\right)}>b-\mu_{1}.$$

#### 3.3.2. Case *E*_1_: criminal-free equilibrium

Let

$$0<\frac{\left(\beta_{3}-\mu_{2}\right)}{\beta_{3}}=\Omega <1.$$

Therefore, *I*_p_=*ΩP*. On substituting *E*_1_ in *Q*, the eigenvalues are

(i) −2*β*_3_*Ω*−*μ*_2_<0
(ii) $-\frac{a^{\prime }P(1-\Omega (1-r))}{\left(1-cP\right)}-\mu_{1}<0\;\mathrm{and}$
(iii) $b-\frac{a^{\prime }P(1-\Omega +r\Omega )}{\left(1-cP\right)}-\mu_{1}.$

Hence the condition for stability for the criminal-free equilibrium is

3.6 $$\frac{a^{\prime }P(1-\Omega (1-r))}{\left(1-cP\right)}>b-\mu_{1}.$$

#### 3.3.3 Hopf bifurcations for *E*_0_ and *E*_1_

It must be noted that

$$Q=\left[\begin{array}{cc} -\frac{a^{\prime }(P-I_{p}+rI_{p})}{\left(1-cP\right)}-\mu_{1} & 0\\ \frac{\beta_{2}(P-I_{p})}{P} & -\frac{2\beta_{3}I_{p}}{P}-\mu_{2} \end{array}\right]$$

at *E*_0_ and *E*_1_, where *I*_p_=0 for *E*_0_. The matrix has a characteristic polynomial of

$$\begin{array}{r} X^{2}+\left(\mu_{1}+\mu_{2}+\frac{a^{\prime }(P-I_{p}+rI_{p})}{\left(1-cP\right)}+\frac{2}{P}\beta_{3}I_{p}\right)X+\left(\mu_{2}+\frac{2}{P}\beta_{3}I_{p}\right)\left(\mu_{1}+\frac{a^{\prime }(P-I_{p}+rI_{p})}{\left(1-cP\right)}\right). \end{array}$$

In order to obtain complex conjugate pairs of eigenvalues at these equilibria,

$$\left(\mu_{1}+\mu_{2}+\frac{a^{\prime }(P-I_{p}+rI_{p})}{\left(1-cP\right)}+\frac{2\beta_{3}I_{p}}{P}\right)=0$$

and

$$\left(\mu_{2}+\frac{2}{P}\beta_{3}I_{p}\right)\left(\mu_{1}+\frac{a^{\prime }(P-I_{p}+rI_{p})}{\left(1-cP\right)}\right)>0.$$

This is not possible so there will be no Hopf bifurcations for the no-criminals equilibria.

#### 3.3.4. Case *E*_2_: core gang-free and corrupted police-free equilibrium

On substituting *E*_2_ in *Q*, the eigenvalues are

(i) $\frac{ha^{\prime }S_{g}P}{{\left(hS_{g}-Pc+1\right)}^{2}}-\frac{S_{g}}{K},$
(ii) *β*_1_−*b* and
(iii) −*μ*_2_.

Hence, the conditions for stability for the core gang-free and corrupted police-free equilibrium are

3.7 $$\frac{ha^{\prime }P}{{\left(hS_{g}-Pc+1\right)}^{2}}-\frac{1}{K}<0\;\text{and}\;\beta_{1}<b.$$

#### 3.3.5. Case *E*_3_: core gang-free equilibrium

On substituting *E*_3_ in *Q*, the eigenvalues are:

(i) $\frac{a^{\prime }hPS_{g}(\mu_{2}+r\beta_{3}-r\mu_{2})}{\beta_{3}{\left(hS_{g}-Pc+1\right)}^{2}}-\frac{S_{g}}{K},$
(ii) *β*_1_−*b* and
(iii) $-\frac{2\beta_{3}I_{p}}{P}-\mu_{2}.$

Hence, the conditions for stability for the core gang-free equilibrium are

3.8 $$\frac{a^{\prime }hP(\mu_{2}+r\beta_{3}-r\mu_{2})}{\beta_{3}{\left(hS_{g}-Pc+1\right)}^{2}}<\frac{1}{K}\;\mathrm{and}\;\beta_{1}<b.$$

#### 3.3.6. Hopf bifurcations for *E*_2_ and *E*_3_

It must be noted that

$$Q=\left[\begin{array}{cc} \beta_{1}-b & 0\\ \frac{\beta_{2}(P-I_{p})}{P} & -\frac{2\beta_{3}I_{p}}{P}-\mu_{2} \end{array}\right]$$

at *E*_2_ and *E*_3_, has a characteristic polynomial

$$X^{2}+\left(b-\beta_{1}+\mu_{2}+\frac{2}{P}\beta_{3}I_{p}\right)X+\left(\mu_{2}+\frac{2}{P}\beta_{3}I_{p}\right)(b-\beta_{1}).$$

In order to obtain complex conjugate pairs of eigenvalues at these equilibria,

3.9 $$\left(b-\beta_{1}+\mu_{2}+\frac{2}{P}\beta_{3}I_{p}\right)=0$$

and

3.10 $$\left(\mu_{2}+\frac{2}{P}\beta_{3}I_{p}\right)(b-\beta_{1})>0.$$

Note that (3.9) is not possible as *β*_1_<*b*.

#### 3.3.7 Case *E*_4_: positive interior equilibrium with coexistence

The expressions for $\tilde{E}=({\tilde{S\;}\;\;}_{g},{\tilde{I\;}\;\;}_{g},{\tilde{I\;}\;\;}_{g})$ are given by (3.1) and (3.2). Owing to the complicated nature of these expressions, stability analysis will be done numerically using Matlab to calculate eigenvalues for this state.

## 4. Discussion of results

Disease and predation play an important role in regulating populations. We have investigated the effects of corruption and predation on the gang and police populations using an eco-epidemiological model. Eco-epidemiology is a relatively recent addition to the field of mathematical biology [29] combining infectious disease and ecological dynamics. There are three basic types of eco-epidemiological models: models incorporating disease spread in the prey [30,31]; models of disease spread in the predators [32]; and models of contagion in both predator and prey populations [33,34]. One of the main differences between this model and other conventional eco-epidemiological models is that the growth rate for police officers does not depend on the capture rate of gang members. This is because there is a fixed number of police officers entering the system each year, as Police Training Academies can only admit a certain number of recruits.

Estimating the parameters in this model was challenging. This was due to the difficulty in quantifying parameters as well as difficulty in obtaining police information about their operations and accessing crime data, which is not an uncommon problem faced by crime modellers [35]. As the issue of corruption in the police is an especially sensitive one, we were unable to get any official data from the police and relied heavily on anecdotal and media reports. The values for the parameters used are shown in table 3. Some of these parameter values were taken from a previously published paper treating the spread of gangs as an infectious disease [16].

**Table 3.** Parameter values used in numerical simulations.

parameter	value	parameter	value	parameter	value
*b*	0.2000	*r*	0.7500	*β*_2_	0.1050
*μ*_1_	0.1200	*λ*	1.500	*β*_3_	0.0525
*μ*_2_	0.0476	*t*_h_	0.0100	*a*	0.0010
*μ*_3_	0.2000	*U*	10.00	*c*	0.0010
*β*_1_	0.2100	*c*	0.0010	*K*	50000

Using these parameter values, cases *E*_0_−*E*_4_ were checked for stability. Cases *E*_0_, *E*_1_, *E*_2_ and *E*_3_ were found to be unstable for these parameter values. This means that this system cannot tend to one of the states of having no core gang members and tends instead to the stable coexistence endemic equilibrium *E*_4_ where

$$S_{g}^{*}=2124,\;I_{g}^{*}=106,\;S_{p}^{*}=123,\;I_{p}^{*}=177,\;Z^{*}=395.$$

The eigenvalues are: −0.0413,−0.2048 and −0.1440. As the eigenvalues are all negative, the system is stable.

Because we wish to determine whether it is possible for the police officers to eliminate gang members even when they themselves are diseased or corrupt, the model is analysed for bifurcations.

### 4.1 Bifurcation analysis

To consider the influence of parameter values on the long term behaviour, we are interested in those critical values at which the asymptotic behaviour changes qualitatively when the critical values are passed. When a parameter is varied, there is the possibility that nothing interesting happens and there is only a quantitatively different behaviour—shifted equilibria, etc. On the other hand, the system may change suddenly and exhibit a very different behaviour. The tipping point at which this happens is known as the bifurcation point. Matcont is used for the bifurcation analysis. Bifurcation points were found for *b*,*β*_1_,*μ*_1_,*a* and *P* (table 4). Changing the other parameters led to a quantitative change, but qualitatively the system’s behaviour remained the same.

**Table 4.** Bifurcation points for the parameter values used in the model.

parameter	range	equilibrium state of system
*β*_1_	0<*β*_1_<0.2	*E*_3_
	*β*_1_>0.2	*E*_4_
*μ*_1_	0<*μ*<0.158143	*E*_4_
	*μ*>0.158143	*E*_1_
*P*	0<*P*<450.28143	*E*_4_
	*P*>450.28143	*E*_1_
*a*	0<*a*<0.0019	*E*_4_
	*a*>0.0019	*E*_1_
*b*	0<*b*<0.16186	*E*_1_
	0.16186<*b*<0.21	*E*_4_
	*b*>0.21	*E*_3_

As the parameter values used were such that *β*_3_>*μ*_2_ and there were no bifurcations with respect to these parameters, then the system tended to either the criminal-free equilibrium *E*_1_, the core gang-free equilibrium *E*_3_ , where *I*_g_=*P*(*β*_3_−*μ*_2_)/*β*_3_ or the coexistence equilibrium *E*_4_ , where *I*_p_≠0 depending on the stability conditions for these equilibria.

#### 4.1.1 Bifurcations with respect to *β*_1_

**Figure 1.**
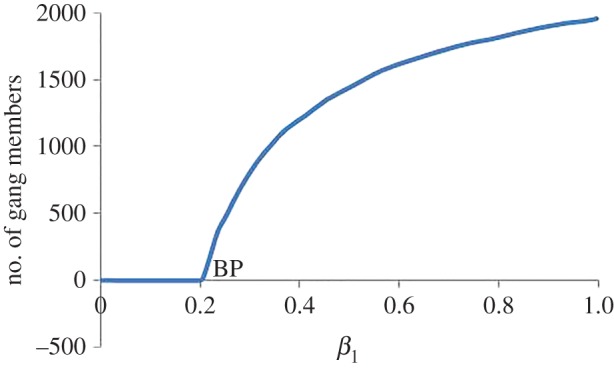
Bifurcation diagram for *β*_1_ illustrating the transcritical bifurcation at *β*_1_=0.2. For values of *β*_1_<0.2, the system tends to the core gang-free equilibrium *E*_3_ with no committed core gang members. Otherwise, the system tends to the coexistence equilibrium *E*_4_.

Figure 1 shows the bifurcation diagram with respect to *β*_1_. There is a transcritical bifurcation at *β*_1_=0.2. According to the condition for stability (3.8), when *β*_1_<*b*=0.2, the system will tend to the core gang-free equilibrium *E*_3_. For *β*_1_>0.2, the system tends to the coexistence equilibrium *E*_4_.

We illustrate the stability of the *E*_4_ coexistence equilibrium solution at *β*_1_=0.71 (as seen in figure 1) using (3.3) with a small perturbation of 0.0001. The *E*_4_ coexistence equilibrium solution values are

$$S_{g}^{*}=2124,\;I_{g}^{*}=106,\;S_{p}^{*}=123,\;I_{p}^{*}=177.$$

**Figure 2.**
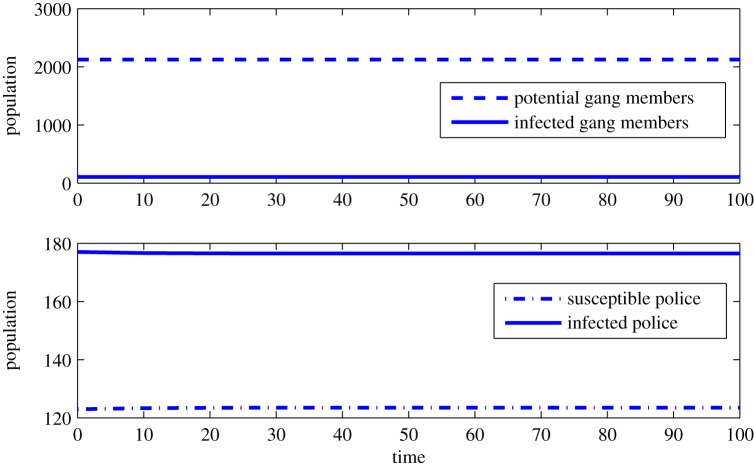
Time series plot of system populations at *β*_1_=0.71 illustrating the stable coexistence equilibrium *E*_4_.

Figure 2 shows that when this stable equilibrium is perturbed, the system returns to the coexistence state. A similar numerical stability analysis may be done with the other bifurcation parameters.

#### 4.1.2 Bifurcations with respect to *μ*_1_

**Figure 3.**
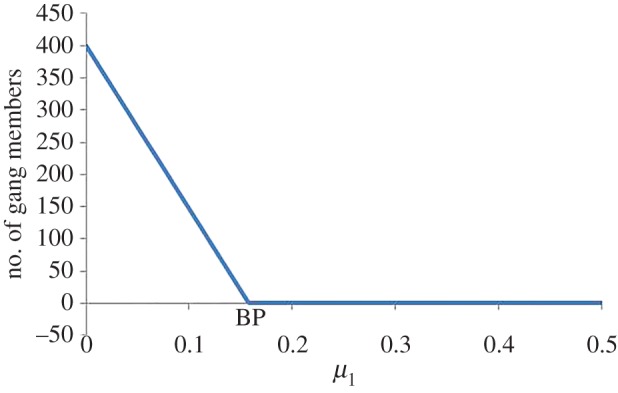
Bifurcation diagram for *μ*_1_ showing the transcritical bifurcation at *μ*_1_= 0.158143. For *μ*_1_<0.158143, the system tends to the coexistence equilibrium *E*_4_ with all populations present. Otherwise, the system tends to the criminal-free equilibrium *E*_1_ with no criminal gang members.

Figure 3 shows a transcritical bifurcation point at *μ*_1_=0.158143. As *β*_3_>*μ*_2_, and *β*_1_>*b*, the system will tend to either the criminal-free equilibrium *E*_1_ or the coexistence equilibrium *E*_4_. For *μ*_1_<0.158143, the system tends to the coexistence equilibrium *E*_4_. The bifurcation point may be obtained from (3.6). The condition for stability of *E*_1_ is

$$\mu_{1}>b-\frac{a^{\prime }P(1-\Omega +r\Omega )}{\left(1-cP\right)}.$$

Upon substituting parameter values, this corresponds to *μ*_1_>0.158143.

#### 4.1.3 Bifurcations with respect to *P*, *a* and *b*

**Figure 4.**
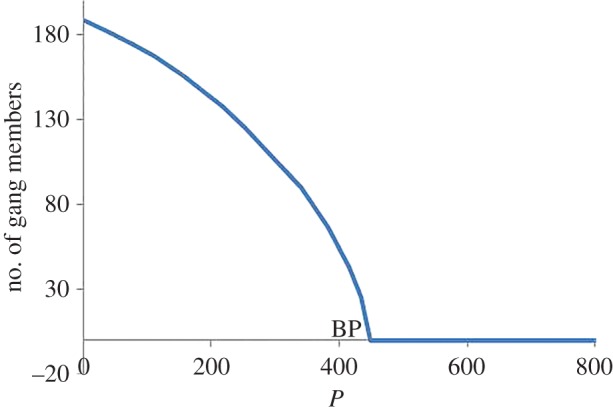
Bifurcation diagram for *P* showing the transcritical bifurcation at *P*= 450.28143. For *P*>450.28143, the system tends to the criminal-free equilibrium *E*_1_ with no criminal gang members. Otherwise, the system tends to the coexistence equilibrium *E*_4_.

A similar analysis may be done for *P* and *a*. As shown in figure 4, for values *P*>450.28143, it is possible to reduce the number of gang members to zero with the system tending to the criminal-free equilibrium *E*_1_, otherwise the system tends to the coexistence equilibrium *E*_4_. The bifurcation point may be obtained from (3.6), the conditions for stability of the criminal-free equilibrium *E*_1_ where

$$P>\frac{\left(b-\mu_{1}\right)}{a^{\prime }(1-\Omega (1-r))+(b-\mu_{1}))}.$$

Numerical analysis showed a bifurcation at *a*=0.0019, so that for values of *a*>0.0019, the system tended to the criminal-free equilibrium *E*_1_. (3.6) may be used to calculate the bifurcation point where for stability of *E*_1_

$$a>\frac{U(b-\mu_{1})(1-cP)}{P(1-\Omega (1-r))}.$$

Corruption destabilizes all aspects of the criminal justice system. Improving the criminal justice system can help reduce corruption, both directly by detecting it and indirectly because better law enforcement makes corruption more expensive and more difficult. Looking at all aspects of the criminal justice system—detection, apprehension, prosecution and conviction—would lead to an unduly complex model, so the first stage in the process is looked at—apprehension of gang members. This is represented by the search rate *a* and the number of police officers *P*. For values of *a*>0.0019 and *P*>450.28143, the system tended to the criminal-free equilibrium *E*_1_. This means that the criminals may be eradicated from a coexistence state by a suitably high predation rate *a* on them by the predators/police officers or by a certain number of police officers *P*. There is a limit on the search rate, so the model can be used to get the more efficient search rate or the number of police officers needed so as to make maximum use of resources at a minimal cost.

When *b* is varied, all the populations coexist with values given by *E*_4_ for the range of values 0.16186<*b*<0.21 with a maximum at *b*=0.186. We can calculate the bifurcation value for the criminal-free equilibrium *E*_1_ using (3.6)

$$\begin{array}{rl} b & <\frac{a^{\prime }P(1-\Omega (1-r))}{\left(1-cP\right)}+\mu_{1},\\ b & <0.16186. \end{array}$$

For *b*>0.21, the system tends to the core gang-free equilibrium *E*_3_. From (3.8), the conditions for stability are

4.1 $$\frac{a^{\prime }hP(\mu_{2}+r\beta_{3}-r\mu_{2})}{\beta_{3}{\left(hS_{g}-Pc+1\right)}^{2}}<\frac{1}{K}\;\text{and}\;\beta_{1}<b.$$

and

4.2  ⟹ b>0.21

## 5. Conclusion

Internationally, there is widespread anecdotal evidence of the pervasiveness of police officer corruption by gangs. This model considered corruption of police officers by gang members and other corrupt police officers. A modified predator–prey model with transmissible disease in both the police officers (predator) and gang members (prey) was proposed and analysed, with infected gang members being more vulnerable to predation and infected police officers hunting at a reduced rate. We examined the possibility of police control of gang members even with ‘diseased’ police officers.

The dynamics of the model were examined and parameters were identified which when varied would lead to a rapid decrease in the number of gang members. The system had five steady states, four of which contained no core gang members and one where all the populations coexisted. Because we used parameter values where the spread of disease *β*_3_ among the police officers was greater than the death rate of the police officers *μ*_2_, the corrupt police population survived, when they would otherwise become extinct. Bifurcation points were found for *b*,*β*_1_,*μ*_1_,*a* and *P*, the first three of which were related to the creation of the gang member and the others related to the efficiency of the criminal justice system.

In view of our limited access to data and information about the dynamics of gangs and the operational procedures of police, it proved difficult to parametrize the model. Future work could involve working with the police to develop a more realistic model. However, the model in its present form may be used to gain insights into the effects of varying parameters on the behaviour of the nonlinear system.

## Data accessibility

All materials utilized for the estimation of parameters have been stated throughout the body of the paper and included in the reference section. The graphs were produced using Matcont, a Matlab package for numerical bifurcation analysis of ordinary differential equations that is freely available for download at https://sourceforge.net/projects/matcont.

## Appendix A

The quadratic expression can be obtained for *S*_g_ in *E*_2_ and *E*_3_ of the form

$$b_{1}S_{g}^{2}+b_{2}S_{g}+b_{3}=0,$$

where for *E*_2_, the infection-free equilibrium:

$$\begin{array}{rl} b_{1} & =Uh,\\ b_{2} & =U(X-BKh),\\ b_{3} & =KaP-BKUX, \end{array}$$

and for *E*_3_, the core gang-free equilibrium:

$$\begin{array}{rl} b_{1} & =Uh,\\ b_{2} & =U(X-BKh),\\ b_{3} & =Ka\left(P+R\frac{P\left(\beta_{3}-\mu_{2}\right)}{\beta_{3}}\right)-BKUX,\\ \text{where}\;X & =1-cP,R=r-1\;\mathrm{and}\;B=(b-\mu_{1}). \end{array}$$

## Appendix B

For the coexistence equilibrium *E*_4_, the coefficients of the polynomial (3.2) are as follows:

$$\begin{array}{rl} b_{1} & =h\Omega \chi \beta_{3}^{2},\\ b_{2} & =\beta_{2}a^{\prime }R+\chi \beta_{2}\beta_{3}Q-2Ph\Omega \chi \beta W+Bh\Omega \beta_{3},\\ b_{3} & =P\beta_{2}a^{\prime }+P\chi \beta_{2}\beta_{3}-BPh\Omega \beta_{3}+2P^{2}c\chi \beta_{2}\beta_{3}+P^{2}c\chi \beta_{2}\mu_{2}-P^{2}h\Omega \chi \beta_{3}\mu_{2}\\ & \;+(-BPh\Omega -P^{2}h\Omega \chi \mu_{2}+P\chi \beta_{2})W-2P\beta_{2}a^{\prime }R+B\beta_{2}Q,\\ b_{4} & =-3P^{2}\beta_{2}a^{\prime }+P^{2}r\beta_{2}a^{\prime }+P^{2}(Bh\Omega +Pc\chi \beta_{2}-\chi \beta_{2})-2BP\beta_{2}Q+P^{2}\beta_{2}a^{\prime }R-2P^{2}\beta_{2}a^{\prime },\\ b_{5} & =P^{3}\beta_{2}a^{\prime }+BP^{2}\beta_{2}Q,\\ R & =(r-1),\\ B & =b-\mu_{1},\\ W & =(\beta_{3}-\mu_{2}),\\ \chi & =\left(\frac{\beta_{1}}{K\beta_{2}(\beta_{1}-b)}\right),\\ \Omega & =\left(\frac{b}{\beta_{1}-b}+\lambda \right),\\ Q & =(Pc-1), \end{array}$$

which can be solved for *I*_p_.

## Appendix C

The coefficients of the polynomial (3.4) are as follows:

$$\begin{array}{rl} a_{1} & =-A_{1}-A_{5}-A_{8},\\ a_{2} & =A_{8}(A_{1}+A_{5})+A_{1}A_{5}-A_{2}A_{4}-A_{6}A_{7},\\ a_{3} & =A_{6}A_{7}(A_{1}+A_{5})-A_{7}(A_{3}A_{4}+A_{5}A_{6})-A_{8}(A_{1}A_{5}-A_{2}A_{4}). \end{array}$$

and

$$\begin{array}{rl} A_{1} & =b-\frac{2S_{g}}{K}-\frac{\beta_{1}I_{g}^{2}}{{\left(S_{g}+I_{g}\right)}^{2}}-\frac{a^{\prime }(P-I_{p}+rI_{p})(1+\lambda hI_{g}-cP)}{{\left(1+hS_{g}+\lambda hI_{g}-cP\right)}^{2}}-\mu_{1},\\ A_{2} & =b-\frac{S_{g}}{K}-\frac{\beta_{1}S_{g}^{2}}{{\left(S_{g}+I_{g}\right)}^{2}}+\frac{a^{\prime }S_{g}(P-I_{p}+rI_{p})\lambda h}{{\left(1+hS_{g}+\lambda hI_{g}-cP\right)}^{2}},\\ A_{3} & =\frac{a^{\prime }(1-r)S_{g}}{\left(1+hS_{g}+\lambda hI_{g}-cP\right)},\\ A_{4} & =\frac{\beta_{1}I_{g}^{2}}{{\left(S_{g}+I_{g}\right)}^{2}}+\frac{a^{\prime }I_{g}h(P-I_{p}+rI_{p})}{{\left(1+hS_{g}+\lambda hI_{g}-cP\right)}^{2}}-\frac{I_{g}}{K},\\ A_{5} & =\frac{\beta_{1}S_{g}^{2}}{{\left(S_{g}+I_{g}\right)}^{2}}-\frac{2I_{g}}{K}-\frac{S_{g}}{K}-\frac{a^{\prime }(P-I_{p}+rI_{p})(1+hS_{g}-cP)}{{\left(1+hS_{g}+\lambda hI_{g}-cP\right)}^{2}}-\mu_{1},\\ A_{6} & =\frac{a^{\prime }(1-r)I_{g}}{\left(1+hS_{g}+\lambda hI_{g}-cP\right)},\\ A_{7} & =\frac{\beta_{2}(P-I_{p})}{P},\\ A_{8} & =-\frac{\beta_{2}I_{g}}{P}-\frac{2\beta_{3}I_{p}}{P}-\mu_{2},\\ A_{9} & =\frac{a^{\prime }(P-I_{p}+rI_{p})(1+\lambda hI_{g}-cP)}{{\left(1+hS_{g}+\lambda hI_{g}-cP\right)}^{2}}-\frac{a^{\prime }I_{g}h(S_{p}+rI_{p})}{{\left(1+hS_{g}+\lambda hI_{g}-cP\right)}^{2}},\\ A_{10} & =\frac{-a^{\prime }S_{g}(P-I_{p}+rI_{p})\lambda h}{{\left(1+hS_{g}+\lambda hI_{g}-cP\right)}^{2}}+\frac{a^{\prime }(P-I_{p}+rI_{p})(1+hS_{g}-cP)}{{\left(1+hS_{g}+\lambda hI_{g}-cP\right)}^{2}},\\ A_{11} & =\frac{a^{\prime }(r-1)S_{g}}{\left(1+hS_{g}+\lambda hI_{g}-cP\right)}+\frac{a^{\prime }(r-1)I_{g}}{\left(1+hS_{g}+\lambda hI_{g}-cP\right)}. \end{array}$$

## References

[1] Rajs J, Harm T, Brodin U 1987 A statistical model examining repetitive criminal behavior in acts of violence. *Am. J. Forensic Med. Pathol.* 8, 103–106. (doi:10.1097/00000433-198708020-00003) 3605002 10.1097/00000433-198708020-00003

[2] Osgood DW 2010 Statistical models of life events and criminal behavior. In *Handbook of quantitative criminology* (eds RA Piquero, D Weisburd), pp. 375–396. New York, NY: Springer.

[3] Malleson N, Evans A 2014 Agent-based models to predict crime at places. In *Encyclopedia of criminology and criminal justice*, pp. 41–48. New York, NY: Springer.

[4] Bosse T, Gerritsen C, Hoogendoorn M, Jaffry S, Treur J 2011 Agent-based vs. population-based simulation of displacement of crime: a comparative study. *Web Intell. Agent Syst.* 9, 147–160.

[5] Jones PA, Brantingham PJ, Chayes LR 2010 Statistical models of criminal behavior: the effects of law enforcement actions. *Math. Mod. Meth. Appl. Sci.* 20(Suppl.), 1397–1423. (doi:10.1142/S0218202510004647)

[6] Bosse T, Gerritsen C 2010 Social simulation and analysis of dynamics of criminal hot spots. *J. Artif. Soc. Soc. Simulat.* 13, 5 (doi:10.18564/jasss.1498)

[7] Perc M, Donnay K, Helbing D 2013 Understanding recurrent crime as system – immanent collective behavior. *PLoS ONE* 8, e76063 (doi:10.1371/journal.pone.0076063) 24124533 10.1371/journal.pone.0076063 PMC3790713

[8] Hegemann RA, Smith LM, Barbaro AB, Bertozzi AL, Reid SE, Tita GE 2011 Geographical influences of an emerging network of gang rivalries. *Phys. A Stat. Mech. Appl.* 390, 3894–3914. (doi:10.1016/j.physa.2011.05.040)

[9] Groff ER 2007 Simulation for theory testing and experimentation: an example using routine activity theory and street robbery. *J. Quant. Criminol.* 23, 75–103. (doi:10.1007/s10940-006-9021-z)

[10] Malleson N, Heppenstall A, See L 2010 Crime reduction through simulation: an agent-based model of burglary. *Comp. Environ. Urban Sys.* 34, 236–250. (doi:10.1016/j.compenvurbsys.2009.10.005)

[11] Barbaro A 2015 A place for agent based models: comment on ‘Statistical physics of crime: a review’ by M.R. D’Orsogna and M. Perc. *Phys. Life Rev.* 12, 24–25. (doi:10.1016/j.plrev.2015.01.022) 25638447 10.1016/j.plrev.2015.01.022

[12] D’Orsogna MR, Perc M 2015 Statistical physics of crime: a review. *Phys. Life Rev.* 12, 1–21. (doi:10.1016/j.plrev.2014.11.001) 25468514 10.1016/j.plrev.2014.11.001

[13] Brown C 1995 *Serpents in the sand: essays on the nonlinear nature of politics and human destiny*. Ann Arbor, MI: University of Michigan Press.

[14] Campbell M, Ormerod P 1997 Social interaction and the dynamics of crime. Technical Report. London, UK: Volterra Consulting Ltd.

[15] Ormerod P, Mounfield C, Smith L 2001 Nonlinear modelling of burglary and violent crime in the UK. In *Modelling crime and offending: recent developments in England and Wales* (ed. C Lewis), p. 80. London, UK: Research, Development and Statistics Directorate.

[16] Sooknanan J, Bhatt B, Comissiong DMG 2012 Life and death in a gang – a mathematical model of gang membership. *J. Math. Res.* 4, 10–27. (doi:10.5539/jmr.v4n4p10)

[17] Sooknanan J, Bhatt B, Comissiong DMG 2013 Catching a gang: a mathematical model of the spread of gangs in a population. *IJPAM* 83, 25–44. (doi:10.12732/ijpam.v83i1.4)

[18] Vargo L 1966 A note on crime control. *Bull. Math. Biol.* 28, 375–378. (doi:10.1007/bfo2476819) 10.1007/BF02476819 5970934

[19] Sooknanan J, Bhatt B, Comissiong DMG 2012 Criminals treated as predators to be harvested: a two prey one predator model with group defense, prey migration and switching. *J. Math. Res.* 4, 92–106. (doi:10.5539/jmr.v4n4p92)

[20] Nuño JC, Herrero MA, Primicerio M 2008 A triangle model of criminality. *Phys. A Stat. Mech. Appl.* 387, 2926–2936. (doi:10.1016/j.physa.2008.01.076)

[21] Newburn T, Webb B 1999 *Understanding and preventing police corruption: lessons from the literature*. London, UK: Policing and Reducing Crime Unit. Research, Development and Statistics Directorate.

[22] Chene M 2010 Anti-corruption and police reform. *Transparency Int.* U4 247, 1–10.

[23] Hults R 2008 *Comprehensive community reanimation process*. Hazleton, PA: Urban Dynamics Inc.

[24] Hwang TW, Kuang Y 2003 Deterministic extinction effect of parasites on host populations. *J. Math. Biol.* 46, 17–30. (doi:10.1007/s00285-002-0165-7) 12525933 10.1007/s00285-002-0165-7

[25] Jeschke JM, Kopp M, Tollrian R 2002 Predator function responses: discriminating between handling and digesting prey. *Ecol. Mono.* 72, 95–112. (doi:10.1890/0012-9615(2002)072[0095:PFRDBH]2.0.CO;2)

[26] Abrams PA, Ginzburg LR 2000 The nature of predation: prey dependent, ratio dependent or neither? *Trends Ecol. Evol.* 15, 337–341. (doi:10.1016/S0169-5347(00)01908-X) 10884706 10.1016/s0169-5347(00)01908-x

[27] Skalski GT, Gilliam JF 2001 Functional responses with predator interference: viable alternatives to the Holling type II model. *Ecology* 82, 3083–3092. (doi:10.1890/0012-9658(2001)082[3083:FRWPIV]2.0.CO;2)

[28] Beddington JR 1975 Mutual interference between parasites or predators and its effect on searching efficiency. *J. Anim. Ecol.* 44, 331–340. (doi:10.2307/3866)

[29] Su M, Wang H 2015 Modeling at the interface of ecology and epidemiology. *Comput. Ecol. Softw.* 5, 367–379.

[30] Venturino E 1994 The influence of diseases on Lotka-Volterra systems. *Rocky MT. J. Math.* 24, 381–402. (doi:10.1216/rmjm/1181072471)

[31] Hethcote HW, Wang W, Han L, Ma Z 2004 A predator prey model with infected prey. *Theor. Pop. Biol.* 66, 259–268. (doi:10.1016/j.tpb.2004.06.010) 15465126 10.1016/j.tpb.2004.06.010

[32] Venturino E 2016 Ecoepidemiology: a more comprehensive view of population interactions. *Math. Model. Nat. Phenom.* 11, 49–90. (doi:10.1051/mmnp/201611104)

[33] Hsieh YH, Hsiao CK 2008 Predator–prey model with disease in both populations. *Math. Med. Biol.* 25, 247–266. (doi:10.1093/imammb/dqn017) 18701422 10.1093/imammb/dqn017 PMC7108608

[34] Chaudhuri S, Costamagna A, Venturino E 2012 Epidemic spreading in predator–prey systems. *Int. J. Comput. Math.* 89, 561–584. (doi:10.1080/00207160.2011.648183)

[35] Felson M 2010 What every mathematician should know about modelling crime. *Eur. J. Appl. Math.* 21, 275–281. (doi:10.1017/S0956792510000070)

